# How do amino acid substitutions affect the amyloidogenic properties and seeding efficiency of prion peptides

**DOI:** 10.1007/s00726-013-1522-0

**Published:** 2013-06-05

**Authors:** Chi-Chen Chuang, Tai-Yan Liao, Eric H.-L Chen, Rita P.-Y. Chen

**Affiliations:** 1 Department of Biochemical Science and Technology, National Taiwan University, Taipei, 106 Taiwan, ROC; 2 Institute of Biological Chemistry, Academia Sinica, No. 128, Sec. 2, Academia Rd, Nankang, Taipei, 115 Taiwan, ROC

**Keywords:** Prion, Amyloid, Fibril, Seeding, Species barrier, Kinetics

## Abstract

The amino acid sequences in the amyloidogenic region (amino acids 108–144) of several mammalian prion proteins were compared and variations were found to occur at residues 109 (M or L), 112 (M or V), 129 (M, V, or L), 135 (N or S), 138 (M, L, or I), 139 (M or I), and 143 (N or S). Using the bovine PrP peptide (residues 108–144 based on the numbering of the human prion protein sequence) as a control peptide, several peptides with one amino acid differing from that of the bovine PrP peptide at residues 109, 112, 135, 138, 139, or 143 and several mammalian PrP peptides were synthesized, and the effects of these amino acid substitutions on the amyloidogenic properties of these peptides were compared and discussed on the basis of the chemical and structural properties of amino acids. Our results showed that the V112M substitution accelerated nucleation of amyloidogenesis, while the N143S and I139M substitutions retarded nucleation. These effects tended to cancel each other out when two substitutions with opposite effects were present on the same peptide. Moreover, acceleration or inhibition of nucleation was not necessarily correlated with effect on seeding efficiency. Using amyloid fibrils prepared from the bovine PrP peptide as seeds, the seeding efficiency for the monomer peptides with the M129L, S135N, N143S, or I139M substitution was decreased compared to that for bPrP peptide. Of all the mammalian peptides used in this study, the dog, mule deer, and pig PrP peptides had the lowest seeding efficiencies.

**Electronic supplementary material:**

The online version of this article (doi:10.1007/s00726-013-1522-0) contains supplementary material, which is available to authorized users.

## Introduction

Prion diseases are fatal transmissible neurodegenerative diseases affecting various mammals, including the sheep, goat, cattle, human, mule deer, elk, cat, and mink (Chesebro 2003; Prusiner 1998; Aguzzi and Polymenidou 2004; Collinge 2001; Palmer and Collinge 1997; Imran and Mahmood 2011). In 1982, Prusiner and his colleagues (Prusiner et al. (1982), Prusiner (1982) and Bolton et al. (1982)) purified the scrapie agent and coined a new term “prion” to describe the proteinaceous properties of the infectious material. A few years later, the gene encoding the prion protein was cloned (Oesch et al. 1985; Basler et al. 1986) and prion protein was found to be constitutively expressed on the cell surface (Stahl et al. 1987). However, in addition to the cellular form, denoted PrP^C^, the same protein can exist as another isoform, denoted PrP^Sc^, which is the main component of the infectious prion and the culprit of prion disease transmission (Basler et al. 1986). Administration of prion by the intracerebral, intraperitoneal, intravenous, subcutaneous, intraocular, intraspinal, or oral route transmits the disease from one animal to another and hence prion diseases are called transmissible spongiform encephalopathies (TSEs). Transmission within the same species is quite efficient, while transmission from one species to another is very much less. The transmission barrier is reflected in a prolonged incubation time, and repeated passages are often required for prion “adaption” in a new species (Kimberlin and Walker 1978). The so-called “species barrier” in prion transmission is an important and unsolved issue in prion studies.

**Fig. 1:**
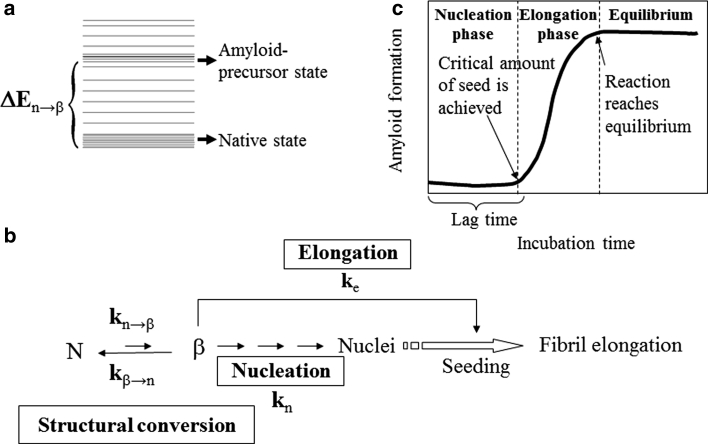
Schematic diagram showing nucleation-dependent polymerization. For a protein to form amyloid, it must be able to undergo structural conversion to a ready-to-stack “amyloid-precursor state”. **a** Energy diagram for the two conformational states; ∆E_n→β_, denotes the energy difference between the native state and the amyloid-precursor state. **b** The process of nucleation-dependent polymerization. *k*_n→β_ is the rate constant for the structural conversion from the native state to the amyloid-precursor state, *k*_n_ is the nucleation rate constant, and *k*_e_ is the elongation rate constant. **c** Time course of amyloidogenesis. For spontaneous amyloidogenesis, the slowest step is nucleus formation. Nuclei accumulate until a critical amount is reached that allows elongation to occur. The time before elongation commences is called the lag time. The elongation process continues until the concentration of protein monomer in the amyloid-precursor state is equal to the dissociation constant for the elongation reaction and equilibrium is reached

The structural conversion from the normal cellular form, PrP^C^, to the disease-causing form, PrP^Sc^, is the key event in prion formation. PrP^Sc^ is a special form of protein aggregate that is rich in cross-β structure called amyloid. Although the structure of PrP^Sc^ remains elusive due to its aggregation nature, synthetic prion peptides consisting of residues 106–126, 127–143, 106–147, 90–145, 171–193 (helix 2), or 199–226 (helix 3) are able to form amyloid fibrils in vitro (Tagliavini et al. 1993; Zhang et al. 1995; Kuwata et al. 2003; Yamaguchi et al. 2008; Walsh et al. 2009; Lin et al. 2010). The amyloid formation process might be explained by a nucleation-dependent polymerization model (Fig. 1), in which there is a long lag phase during which seeds (nuclei) are formed (Jarrett and Lansbury 1993; Caughey et al. 2009; Aguzzi and Polymenidou 2004; Serio et al. 2000; Harper and Lansbury 1997). Prion is the seed that initiates amyloid propagation in prion disease transmission. The interaction between the incoming monomer and the seed stabilizes the cross-β structure and thus facilitates subsequent amyloid elongation. During the elongation phase, the rate of elongation depends on the elongation rate constant (*k*_e_), the molar concentration of the seed (amount of free ends for fibril propagation), and the concentration of monomer in the “amyloid-precursor state”, which is able to associate with the seed.

**Fig. 2:**
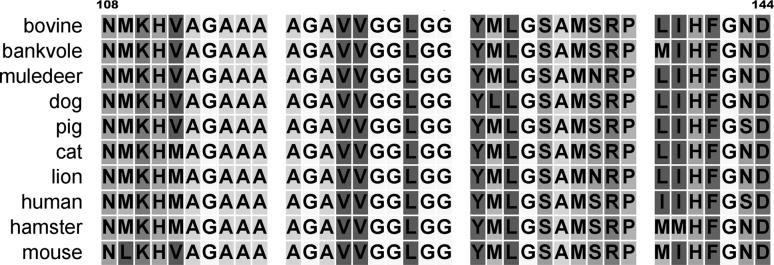
Amino acid sequence comparison of the region corresponding to human prion sequence 108–144 in different mammals. Human PrP has an M/V polymorphism at position-129 and M is shown here

The amino acid sequence determines the three-dimensional structure of a protein. It is known that differences in the amino acid sequence of prion protein do not significantly affect the overall protein structure (Calzolai et al. 2005; Donne et al. 1997; Gossert et al. 2005; Haire et al. 2004; Hornemann et al. 2004; Hosszu et al. 2004; Lysek et al. 2005; Riek et al. 1996; Riek et al. 1998), but markedly affect both its structural conversion from the normal protease-sensitive form (PrP-sen) to the protease-resistant aggregation form (PrP-res) in vitro and the interspecies prion transmission efficiency in vivo (Kocisko et al. 1995; Scott et al. 1989; Vanik et al. 2004; Horiuchi et al. 2000; Priola and Chesebro 1995). In this study, we used a prion peptide (corresponding to human PrP residues 108–144) as a model system to examine how different amino acid sequences influence the amyloidogenic property and seeding efficiency. The region containing residues 108–144 of prion protein was initially predicted to contain two putative helices H1 and H2 (Gasset et al. 1992), but the synthetic peptide PrP (108–144) is known to form a cross-β structure (amyloid) after incubation in solution (Chen et al. 2002; Ho et al. 2009; Liao et al. 2011; Lee and Chen 2007; Gasset et al. 1992). When we compared the PrP (108–144) sequence in different mammals, which may or may not be transfected with TSE, we found sequence variations at position 109 (M/L), 112 (M/V), 129 (M/V/L), 135 (N/S), 138 (M/L/I), 139 (M/I), and 143(N/S) (Fig. 2). The structural and chemical properties of the amino acids M, L, V, I, N, and S are compared in Table 1. We then synthesized several bPrP mutant peptides to examine how amino acid replacement affected the kinetics of fibrillogenesis and the seeding efficiency of this peptide. Circular dichroism spectroscopy was used in this work instead of Thioflavin T binding assay that is commonly used in amyloid study. It is because fluorescence intensity varies a lot among different peptides, making the determination of seeding efficiency very difficult.

**Table 1:** Comparison of the structural and chemical properties of Met, Val, Leu, Ile, Asn, and Ser

	Met	Val	Leu	Ile	Asn	Ser
Branched at β-carbon	−	+	−	+	−	−
Hydrophilicity^a^ (kcal/mol)	−3.87	−0.40	−0.11	−0.24	−12.07	−7.45
Hydrophobicity^a^ (kcal/mol)	−1.41	−3.10	−3.98	−3.98	7.58	4.34
Van der Waals volume^b^ (Å^3^)	124	105	124	124	96	73
β-sheet forming propensity expressed as the ∆∆G (kcal/mol)^c^	−0.46	−0.53	−0.48	−0.56	−0.38	−0.39
α-Helix-forming propensity expressed as the ∆∆G (kcal/mol)^d^	−0.50	−0.14	−0.62	−0.23	−0.07	−0.35
Turn potential^e^	0.57	0.70	0.66	0.59	1.44	1.15

^a^Values are taken from Radzicka and Wolfenden (1988) and Creighton (1984)

^b^Values are taken from Creighton (1984)

^c^Values are taken from Kim and Berg (1993)

^d^Values are taken from O’Neil and DeGrado (1990)

^e^Values are taken from Hutchinson and Thornton (1994)

## Materials and methods

### Peptide synthesis

The prion peptides used were synthesized using the Fmoc-polyamide method (Chen et al. 2001). The N- and C-terminals of the peptides were acetylated or amidated, respectively, to mimic the configuration and charge state in the full-length protein. The peptides were characterized by mass spectrometry after purification, and after lyophilization, were stored at −30 °C.

#### Circular dichroism spectroscopy

The peptides were dissolved at a concentration of 50 μM in 20 mM NaOAc, 140 mM NaCl (pH 3.7) and incubated in Eppendorf tubes quiescently at 25 °C for amyloid fibril formation. At different incubation times, the samples were placed in a 1-mm quartz cuvette and the CD spectra between 200 and 250 nm recorded on a JASCO J-715 spectrometer (JASCO, Japan), with the bandwidth set to 2 nm and a step resolution of 0.05 nm. Two scans were averaged for each sample and the final signal shown in the plots was obtained from the average of several independent repeats. To avoid contamination, a different quartz cuvette was used for each sample, and the same cuvette was used for the same sample for all time points. The lag time was obtained by fitting the kinetic data using the equation: *F* = *A* + *B*/(1 + e^(k*(*t*_1/2_ − *t*))), where *A* is the signal during the lag phase, *B* the signal difference between the lag phase and post-transition plateau, *t* the time, *t*_1/2_ the time required for half-completion of the fibrillization process, and *k* the rate constant of fibril growth (h^−1^), the lag time being equal to *t*_1/2_–2/*k* for each fitted curve.

#### Seed titration experiments

The amyloid fibrils were spun down by centrifugation, re-suspended in distilled water, and fragmented with 20 cycles of intermittent pulses (5 × 0.5 s; 5 s interval between cycles) using an ultrasonic processor (UP100H, Hielscher, USA) equipped with a 1-mm microtip at a power setting of 40 %.

In seed titration experiments, the kinetics of fibril formation were recorded by time-course measurements on a JASCO J-715 spectrometer (JASCO, Japan). The peptide was dissolved at a concentration of 62.5 μM in 25 mM NaOAc and 175 mM NaCl, pH 3.7. The required volume of sonicated seed solution was made to a final volume of 50 μL with distilled water and added to 200 μL of the 62.6 μM peptide monomer solution to give a final monomer concentration of 50 μM. The samples were placed in 1 mm quartz cuvettes and the kinetics of fibril formation recorded every 5 s at 218 nm for 60 min. The trace over the first 2,000 s was linearly fitted to determine the amyloid propagation rate, and the rates in two or three independent experiments were averaged.

#### Transmission electron microscopy

The samples were deposited on carbon-coated 300-mesh copper grids and incubated for 3 min for absorption. Negative staining was performed by staining with 2 % uranyl acetate for 3 min. After drying, the samples were viewed using a Hitachi H-7000 electron microscope.

#### Determination of the thermodynamic solubility of peptides

Different concentrations of PrP peptides (50, 10, 5, 1 μM) were injected into a C18 column (ZORBAX Eclipse column, 4.6 mm × 25 cm, 5 μm, Agilent, USA) by a HPLC system (Agilent USA), then the peptides were eluted using different acetonitrile/water mixtures containing 0.1 % trifluoroacetic acid for the different peptides and detected by UV absorption at 220 nm, then the area under the peak was integrated to generate a standard calibration curve for each peptide. The PrP peptides were dissolved at a concentration of 50 μM in 20 mM NaOAc, 140 mM NaCl (pH 3.7) and incubated at 25 °C for 3 weeks for fibrillization, then the fibril-containing samples were centrifuged for 30 min at room temperature at 13,200*g* in a centrifuge (Eppendorf 5415D, USA) and the supernatant was injected into the same column described above and the concentration of peptide remaining in the supernatant was then determined based on the standard calibration curve.

## Results

**Fig. 3:**
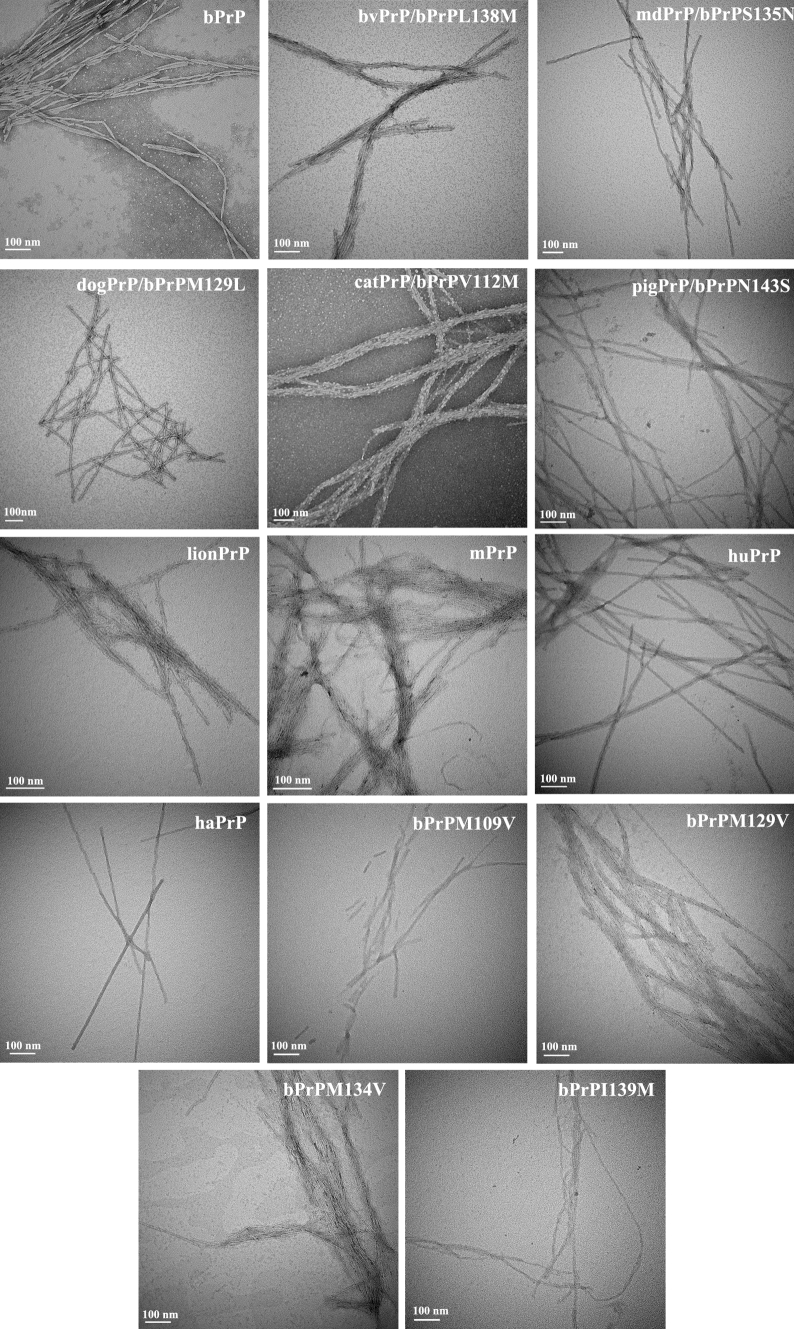
TEM images of the fibrils formed by the different bPrP-based peptides. The *bars* represent 100 nm

Various PrP peptides, synthesized on a solid phase peptide synthesizer, were dissolved in 20 mM NaOAc, 140 mM NaCl (pH 3.7) to a final peptide concentration of 50 μM and incubated at 25 °C. Under these conditions, the peptides initially formed a random coil structure, as shown previously for the hamster peptide (Ho et al. 2009), then gradually transformed into a β structure and associated into amyloid fibrils during incubation, as shown by transmission electron microscopy (TEM) (Fig. 3). The cross-β structural character of amyloid fibrils is reflected by the negative ellipticity at 218 nm of the circular dichroism (CD) spectrum. The time course of amyloidogenesis of the PrP peptides with different mutations was monitored by CD spectroscopy.

### Met → Val or Met → Leu substitution: bPrPM109V, bPrPM129V, bPrPM134V, and bPrPM129L

**Fig. 4:**
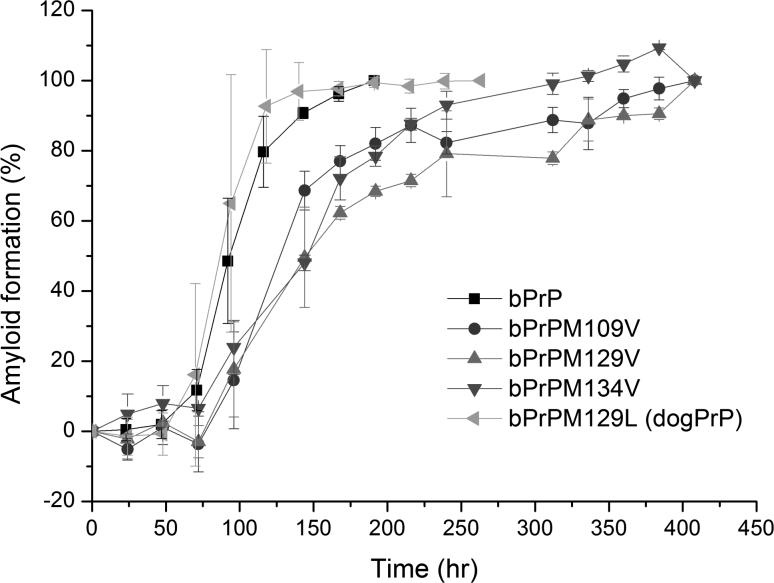
Time course of amyloidogenesis of peptides bPrP, bPrPM109V, bPrPM129V, bPrPM134V, and bPrPM129L monitored by CD spectroscopy

The bPrP peptide contains three methionine residues, M109, M129, and M134, one of which, Met-134, is conserved in all PrP sequences. Most mammalian PrPs also have Met at residues 109 and 129, the exceptions being mouse PrP (Leu at residue 109), dog PrP (Leu at residue 129), and human PrP (Met/Val polymorphism at residue 129). It is known that the M/V polymorphism in human PrP markedly affects the transmission efficiency of variant Creutzfeldt–Jakob disease (Hill et al. 1999). To examine the effect of Met → Val or Met → Leu substitution on amyloidogenesis and seeding efficiency, four single-mutation peptides were prepared; these were bPrPM109V, bPrPM129V, bPrPM134V, and bPrPM129L, with the respective mutations M109 → V, M129 → V, M134 → V, and M129 → L. The negative ellipticity at 218 nm of the CD spectrum was used to monitor the formation of the cross-β structure of amyloid fibrils and the CD signals were normalized using the final signal, taken as 100 % amyloid formation. The kinetics of amyloidogenesis for these peptides are shown in Fig. 4. The lag times for these mutant peptides were similar to that for bPrP, showing that the Met → Val or Leu substitutions did not affect the nucleation step. bPrPM129V and bPrPM134V had slower elongation rates (see their slopes in the elongation phase). We surmise that the Met → Val mutation at residue 129 or 134 might raise the kinetic conversion barrier for bPrPM129V and bPrPM134V from the random coil state to the amyloid-precursor state (decrease the *k*_n→β_). A slower elongation phase has been reported in the kinetic data for human PrP (108–144) peptide with a Met129 → Val substitution (Liao et al. 2011), consistent with our data. A comparison of the structural and chemical properties of Met and Val (Table 1) shows that Val is smaller, more hydrophobic, and has a lower helix-forming propensity and a higher turn-forming potential than Met. We surmise that Val at position 129 or 134 might stabilize the random coil state of these two peptides.

**Fig. 5:**
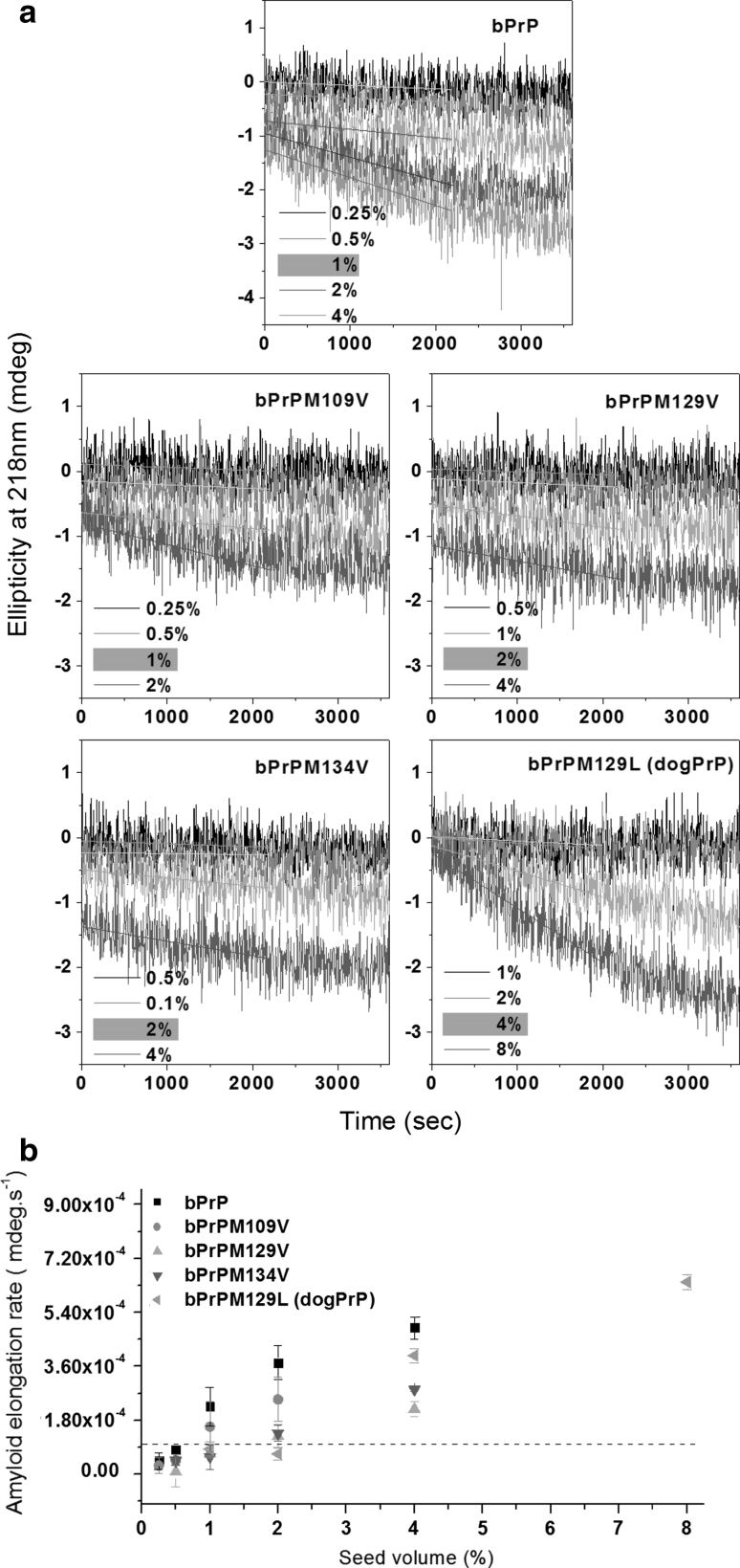
Seeding experiments using bPrP, bPrPM109V, bPrPM129V, bPrPM134V, and bPrPM129L, monitored by time-resolved CD spectroscopy. **a** Different amounts of bPrP seed were added to solutions of peptides bPrP, bPrPM109V, bPrPM129V, bPrPM134V, and bPrPM129L and the kinetics of amyloidogenesis of the mixture monitored at 218 nm. The monomer peptide and the amount of bPrP seed used are indicated in *each panel*. The fibril elongation rate was obtained by linearly fitting the data obtained in the first 2,000 s. The minimum amount of seed required for lag phase-free propagation is highlighted in *yellow*. **b** Plot of initial amyloid elongation rate versus seed amount. The threshold of 1 × 10^−4^ is indicated by the *dashed line*. The minimal amount of bPrP seed required to efficiently seed amyloid formation at the same concentration of peptides bPrP, bPrPM109V, bPrPM129V, bPrPM134V, and bPrPM129L was 1, 1, 2, 2, and 4 %, respectively

The seed titration method is used to evaluate the sequence dependency of seeding efficiency (Liao et al. 2011; Lee and Chen 2007). The kinetics of amyloidogenesis of samples containing the same peptide monomer concentration and different amounts of seed solution, made to the same final volume, were compared in the absence of spontaneous nucleation (the lag time of spontaneous nucleation is much longer than the observation period in the seeding experiments). To control the quality of the seed, homogeneous seed was prepared by ultrasonication of amyloid fibrils formed from the bPrP peptide, as described previously (Lee and Chen 2007), and the amyloidogenesis initiated by seeding was monitored for 1 h by time-resolved CD spectroscopy at 218 nm, the wavelength giving the characteristic signal for cross-β structure formation. The initial amyloid elongation rate was obtained by linear fitting of the ellipticity data within the first 2,000 s. The minimal amount of seed required for immediate amyloidogenesis was arbitrarily and empirically determined as the lowest amount resulting in an initial elongation rate higher than 1 × 10^−4^ m deg/s (the slope in the time-resolved CD spectrum of the peptide solution without fibril formation is always lower than 1 × 10^−4^ and the slope increase is proportional to the increase in seed amount when the slope is higher than 1 × 10^−4^). The seeding efficiency was normalized as the ratio of the minimal amount of seed required for homogeneous seeding to that required for heterogeneous seeding ([seed]_homo_/[seed]_hetero_). For example, the minimal percentage of bPrP seed required to efficiently seed the same concentration of peptides bPrP, bPrPM109V, bPrPM129V, bPrPM134V, and bPrPM129L was 1, 1, 2, 2, and 4 %, respectively (Fig. 5). Using the bPrP seed, the relative seeding efficiencies of peptides bPrP, bPrPM109V, bPrPM129V, bPrPM134V, and bPrPM129L were 1/1, 1/1, 1/2, 1/2, and 1/4, or 1, 1, 0.5, 0.5, and 0.25. bPrPM129L, which corresponds to the dog PrP sequence, had the lowest seeding efficiency among these peptides, suggesting that the Met → Leu substitution at position 129 results in a fourfold increase in the seeding barrier. Whether a Met → Val substitution affects the seeding efficiency of the bPrP fibrils or not depends on the substitution site, as only substitutions in the C-terminal part of the peptide (M129, M134) affected seeding efficiency. Our data are consistent with our previous report (Lee and Chen 2007) that sequence homology between the seed and monomer in the C-terminal part of the peptide is important for seeding when residue 139 of the PrP fibril seed is Ile.

One should be careful to apply the peptide results to full-length prion protein. As mentioned in “Introduction”, many prion peptide segments could form amyloid fibrils, but it is not likely that all these segments are in the amyloid core of the amyloid fibrils formed from full-length prion protein. Lu et al. (2007) have reported the hydrogen/deuterium exchange protection map of huPrP (90–231) fibrils and showed that the region 108–144 is quite flexible compared with the C-terminal region 168–213. Whether the amyloidogenic region 108–144 is really involved in the cross-β structure of the full-length prion fibrils remains to be explored.

### Val, Leu or Ile → Met substitution: bPrPV112M, bPrPL138M, and bPrPI139M

**Fig. 6:**
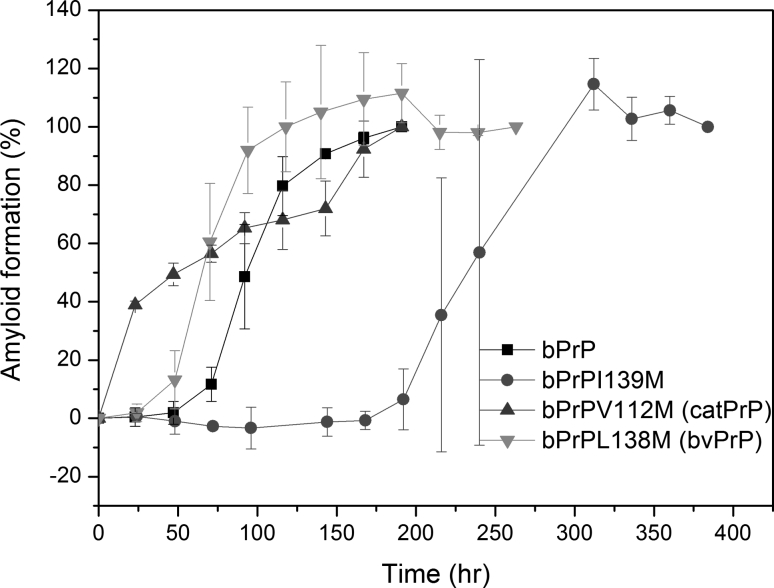
Time course of amyloidogenesis for bPrP, bPrPV112M, bPrPL138M, and bPrPI139M monitored by CD spectroscopy

As shown in Fig. 2, cat, lion, human, and hamster PrPs have Met at position 112 instead of the Val in other animals, while bank vole, mouse, and hamster and human PrPs have Met or Ile at position-138, and other animals have Leu at this position. Most mammal PrPs have Ile at position 139, with the exception of hamster PrP, which has Met at this position. The L138 → I substitution has been reported not to affect amyloidogenesis (Liao et al. 2011). To compare the effects of mutating V/L/I at these positions into M, three single-mutation peptides were prepared: these were bPrPV112M, bPrPL138M, and bPrPI139M, containing, respectively, V112 → M, L138 → M, and I139 → M. The kinetics of amyloidogenesis of these three peptides and bPrP peptide are compared in Fig. 6. The lag time for bPrPI139M was about four times longer than that for bPrP, consistent with a previous report that the mouse PrP peptide, which has Ile at position 139, has a much shorter lag time than a mouse PrP mutant peptide with the Ile 139 to M mutation (Lee and Chen 2007). Our result supports the conclusion that the residue at position 139 markedly affects nucleation. Due to its β-branching property, Ile has a higher β-sheet forming propensity than Met. We surmise that Ile 139 might be involved in cross-β structure formation of the amyloid structure. On the other hand, Leu and Met have a similar β-sheet forming propensity, but Leu has a slightly higher helix-forming propensity and turn-forming potential than Met. The lag time for bPrPL138M was significantly shorter than that for bPrP (*p* < 0.05), suggesting that position 138, together with its neighboring residue Ile 139, are located in the cross-β structure-forming region and that M138 favors nucleation more than L138. Surprisingly, bPrPV112M, which corresponds to the sequence of cat PrP, formed amyloid fibrils very rapidly and its lag time could hardly be measured. M112 might be a key residue responsible for the high prion susceptibility of feline spongiform encephalopathy.

**Fig. 7:**
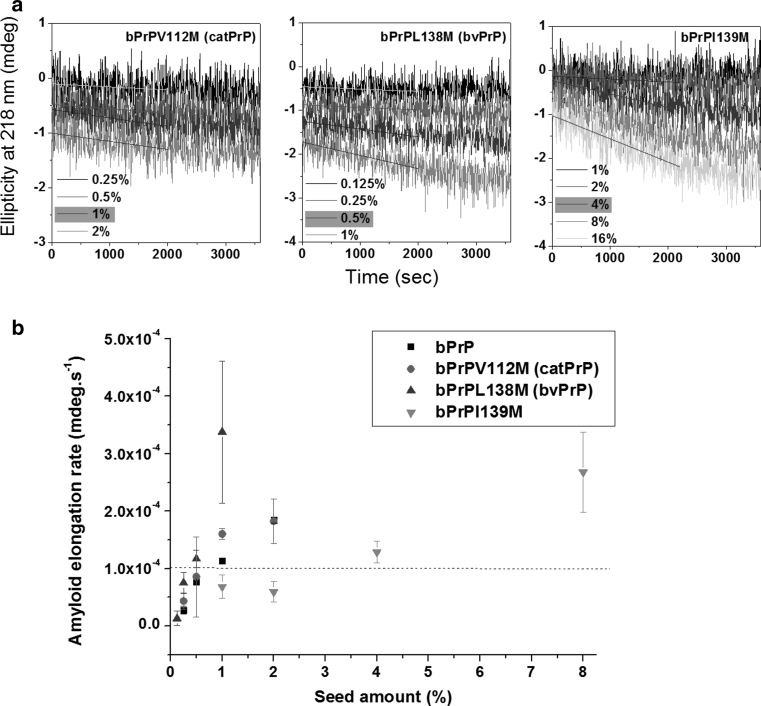
Seeding experiments for bPrP, bPrPV112M, bPrPL138M, and bPrPI139M monitored by time-resolved CD spectroscopy. **a** Different amounts of bPrP seed were added to solutions of peptide bPrPV112M, bPrPL138M, or bPrPI139M and the kinetics of amyloidogenesis of the mixture monitored at 218 nm. The monomer peptide and the amount of seed used are indicated in *each panel*. The fibril elongation rate was obtained by linearly fitting the data from the first 2,000 s. The minimum amount of seed required for lag phase-free propagation is highlighted in *yellow*. **b** Plot of initial amyloid elongation rate versus seed amount. The minimal amount of bPrP seed required to efficiently seed amyloid formation at the same concentration of bPrP, bPrPV112M, bPrPL138M, and bPrPI139M was 1, 1, 0.5, and 4 %, respectively

Using the bPrP seed, the relative seeding efficiencies of peptides: bPrP, bPrPV112 M, bPrPL138M, and bPrPI139M were 1/1, 1/1, 1/0.5, and 1/4 = 1, 1, 2, and 0.25 (Fig. 7). Although the V112 to M mutation greatly accelerated nucleation, it did not affect seeding efficiency. In contrast, the L138 to M mutation had less of an effect on nucleation and increased seeding efficiency. The I139 to M mutation decreased the seeding efficiency by fourfold, consistent with a previous study on mouse/hamster cross-seeding, which showed that, when fibrils derived from mouse PrP peptide (with Ile at position-139) were used as seed, the seeding efficiency for a mouse PrP mutant peptide with the I139 to M mutation was fourfold lower than homogeneous seeding (Lee and Chen 2007).

### Ser → Asn and Asn → Ser substitution: bPrPS135N and bPrPN143S

**Fig. 8:**
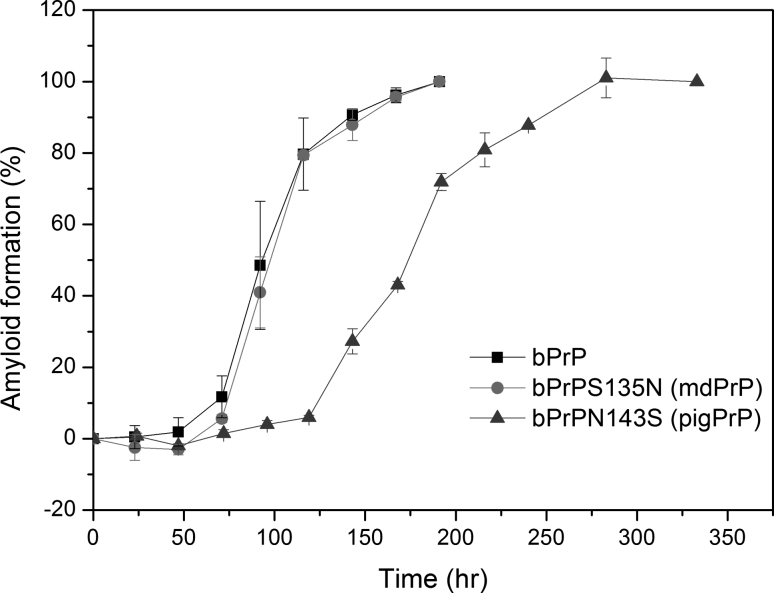
Time course of amyloidogenesis for peptides bPrP, bPrPS135N, and bPrPN143S monitored by CD spectroscopy

As shown in the sequence comparison in Fig. 2, only mule deer and lion PrPs have Asn instead of Ser at position 135 and only pig and human PrPs have Ser instead of Asn at position 143. Gly, Pro, Asn, and Asp are particularly favored in turn structures. How the Asn/Ser substitution affects the amyloidogenic behavior of the PrP peptide was examined by studying two single-mutation peptides bPrPS135N and bPrPN143S containing, respectively, the S135 → N or N143 → S substitution. As shown in Table 1, Asn is more hydrophilic, slightly bigger, and has a higher turn-forming potential and lower helix-forming propensity than Ser. The kinetics of amyloidogenesis of bPrP peptide and peptides bPrPS135N and bPrPN143S are compared in Fig. 8. While the S135 to N mutation did not significantly affect the lag time, the lag time for bPrPN143S was twice as long as that for bPrP, suggesting that the S135 → N mutation does not affect nucleation, while the N143 → S mutation does.

**Fig. 9:**
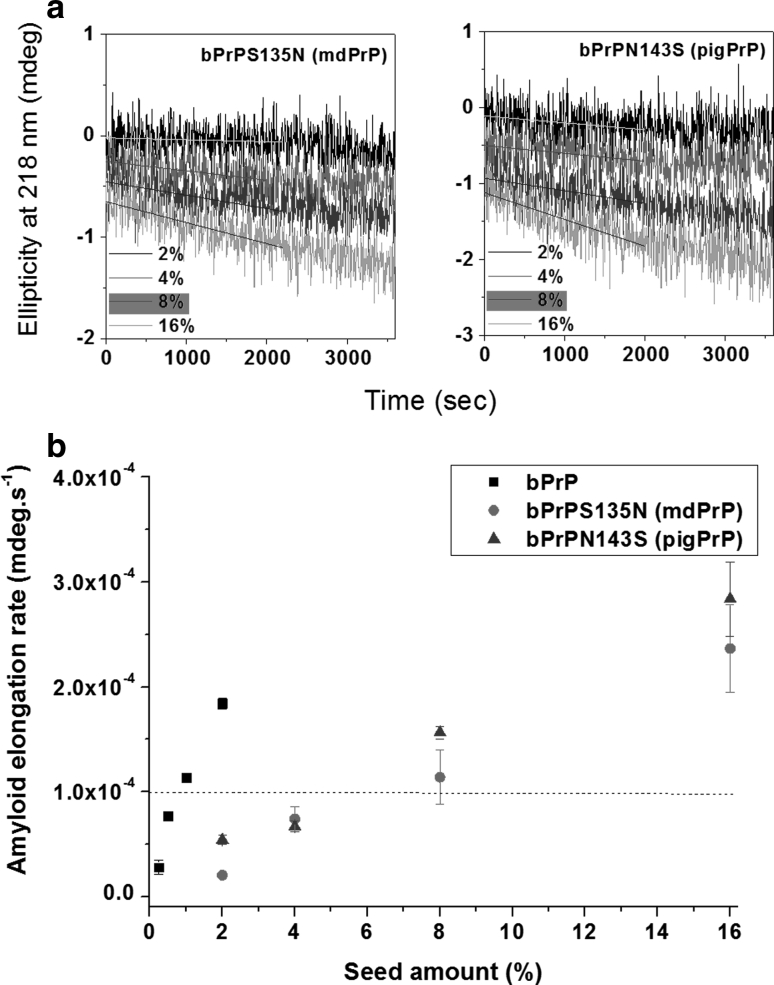
Seeding experiments for peptides bPrP, bPrPS135N, and bPrPN143S monitored by time-resolved CD spectroscopy. **a** Different amounts of bPrP seed were added to a solution of peptide bPrPV112M, bPrPS135N, or bPrPN143S and the kinetics of amyloidogenesis of the mixture monitored at 218 nm. The monomer peptide and the amount of seed used are indicated in *each panel*. The fibril elongation rate was obtained by linearly fitting the data for the first 2,000 s The minimum amount of seed required for lag phase-free propagation is highlighted in *yellow*. **b** Plot of the initial amyloid elongation rate versus seed amount. The minimal amount of bPrP seed required to efficiently seed amyloid formation of the same concentration of bPrP, bPrPS135N, and bPrPN143S was 1, 8, and 8 %, respectively

Using bPrP seed, the relative seeding efficiencies of peptides: bPrP, bPrPS135N, and PrPN143S were 1/1, 1/8, and 1/8 = 1, 0.125, and 0.125 (Fig. 9). The S135N and N143S mutations resulted in an eightfold decrease in heterogeneous seeding efficiency. The amino acid sequence for bPrPS135N is the same as that for the mule deer PrP peptide, while that for bPrPN143S is the same as that for the pig PrP peptide. Our data showed that the mule deer and pig PrP peptides had the largest seeding barrier in the bPrP seeding experiment as a result of the presence of Asn 135 in mule deer and Ser 143 in pig PrP and that the effect of one mutation on the seeding barrier is not necessarily related to its effect on lag time of amyloid fibril formation. Our data also suggested that these two mutations affected the association between the peptide monomer and the pre-existing bPrP seed.

### Other mammalian PrP peptides: lion PrP, mouse PrP, hamster PrP, and human PrP

**Fig. 10:**
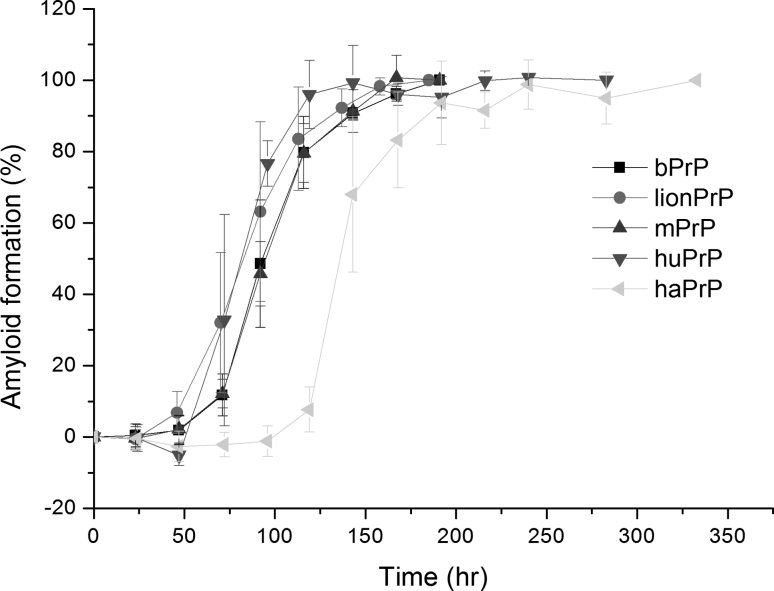
Time course of amyloidogenesis for peptides bPrP, lionPrP, mPrP, haPrP, and huPrP monitored by CD spectroscopy

The data above showed that: (1) Val112 → M accelerates nucleation; (2) Asn143 → Ser and Ile139 → M retard nucleation; and (3) Met129 → Leu, Ser135 → Asn, Asn143 → Ser, and Ile139 → Met decrease the seeding efficiency of bPrP-assisted seeding experiments. As shown in Fig. 1, some mammalian PrP sequences differ from the bovine PrP sequence by more than one residue. Lion and mouse PrP peptides each differ from bPrP peptide by two residues (V112 → M and S135 → N for lionPrP and M109 → L and L138 → M for mPrP), while hamster and human PrP peptides each differ from bPrP peptide by three residues (V112 → M, L138 → M, and I139 → M for haPrP and V112 → M, L138 → I, and N143 → S for huPrP). The lionPrP, mPrP, haPrP, and huPrP peptides were synthesized and their amyloidogenic properties and seeding efficiencies for bPrP seed were examined. As shown in Fig. 10, of these four peptides, only haPrP had a lag time longer than bPrP. As described above, Met 112 is one of the residues promoting amyloid formation and Met 139 one of the residues retarding amyloid formation, and since haPrP has both residues, its lag time of 118.9 h is not as long as that of the bPrPI139M peptide (214.9 h). Similarly, although the N143S substitution has a retardation effect on nucleation, the retardation effect was neutralized by the presence of the V112M substitution in the case of huPrP.

**Fig. 11:**
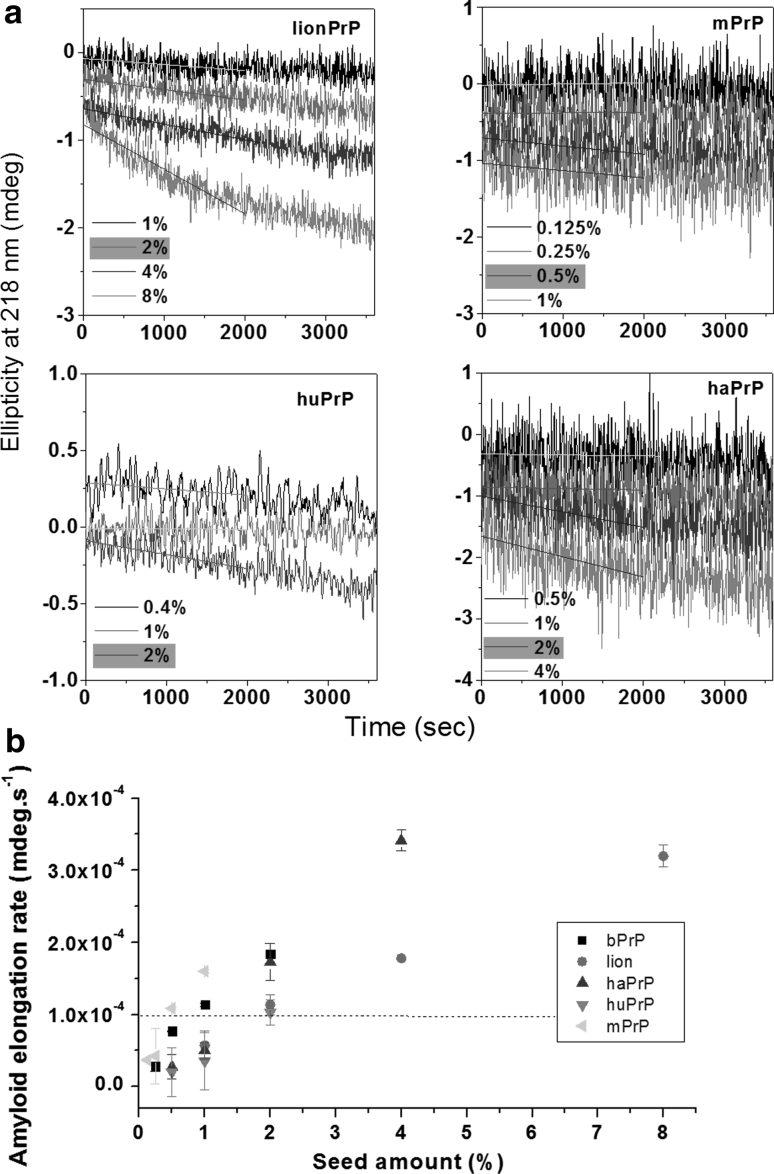
Seeding experiments for bPrP, lionPrP, mPrP, haPrP, and huPrP monitored by time-resolved CD spectroscopy. **a** Different amounts of bPrP seed were added to a solution of peptide lionPrP, mPrP, haPrP, or huPrP and the kinetics of amyloidogenesis of the mixture monitored at 218 nm. The monomer peptide and the amount of seed used are indicated in *each panel*. The fibril elongation rate was obtained by linearly fitting the data for the first 2,000 s. The minimum amount of seed required for lag phase-free propagation is highlighted in *yellow*. **b** Plot of the initial amyloid elongation rate versus seed amount. The minimal amount of bPrP seed required to efficiently seed amyloid formation at the same concentration of bPrP, lionPrP, mPrP, haPrP, and huPrP was 1, 2, 0.5, 2, 2 %, respectively

Using bPrP seed, the relative seeding efficiencies of peptides: bPrP, lionPrP, mPrP, haPrP, and huPrP were 1/1, 1/2, 1/0.5, 1/2, and 1/2 = 1, 0.5, 2, 0.5, and 0.5 (Fig. 11). LionPrP, haPrP, and huPrP had only a slightly lower seeding efficiency than homogeneous seeding.

The amyloidogenic properties of all the PrP peptides used in this study are compared in Table 2. According to the results of single substitutions described above, L138M substitution improves seeding efficiency and S135N, N143S, M129L, and I139M decrease seeding efficiency. When the substitutions of these two groups are present in the same peptide, they tend to cancel each other out. For example, bPrPI139M had a fourfold lower seeding efficiency, and bPrPL138M a twofold higher seeding efficiency, than bPrP homogeneous seeding, but when both substitutions were present in the same peptide (haPrP), the seeding efficiency was only twofold lower than that for bPrP homogeneous seeding.

**Table 2:** Comparison of the amyloidogenic properties of the PrP peptides used in this study

Peptide	Lag time (h)	Remaining monomer concentration (μM)	Normalized seeding efficiency using bPrP fibrils as seeds
bPrP	61.1 ± 10	1.95 ± 0.33	1
One residue difference			
catPrP/bPrPV112M	ND	1.65 ± 0.11	1
dogPrP/bPrPM129L	71.4 ± 12	1.19 ± 0.07***	0.25
bvPrP/bPrPL138M	42.3 ± 13.5*	1.39 ± 0.01**	2
mdPrP/bPrPS135N	72.2 ± 5.5	1.62 ± 0.24	0.125
pigPrP/bPrPN143S	117.8 ± 1.5***	2.79 ± 0.23***	0.125
bPrPM109V	83.3 ± 14.9	1.72 ± 0.06	1
bPrPM129V	57.9 ± 26.3	2.71 ± 0.03***	0.5
bPrPM134V	55.2 ± 13.6	3.29 ± 0.13***	0.5
bPrPI139M	214.9 ± 23.9***	3.25 ± 0.21***	0.25
Two residue difference			
lionPrP (V112M, S135N)	51.2 ± 14.3	1.63 ± 0.06	0.5
mPrP (M109L, L138M)	65.5 ± 6.8	1.09 ± 0.02***	2
Three residue difference			
haPrP (V112M, L138M, I139M)	118.9 ± 4.9***	2.95 ± 0.59***	0.5
huPrP (V112M, L138I, N143S)	61.8 ± 8.4	1.16 ± 0.07***	0.5

For peptides having only one residue different from bPrP, the bPrP-based name is also provided. For peptides with more than one residue different from bPrP, the substitutions are shown in parenthesis

*bPrP* bovine PrP, *bvPrP* bank vole PrP, *mdPrP* mule deer PrP, *mPrP* mouse PrP, *haPrP* hamster PrP, *huPrP* human PrP with Met at residue-129, *ND* not detectable

* *p* < 0.05; ** *p* < 0.01; *** *p* < 0.001 compared to bPrP

### Thermodynamic solubility of the PrP peptides

**Fig. 12:**
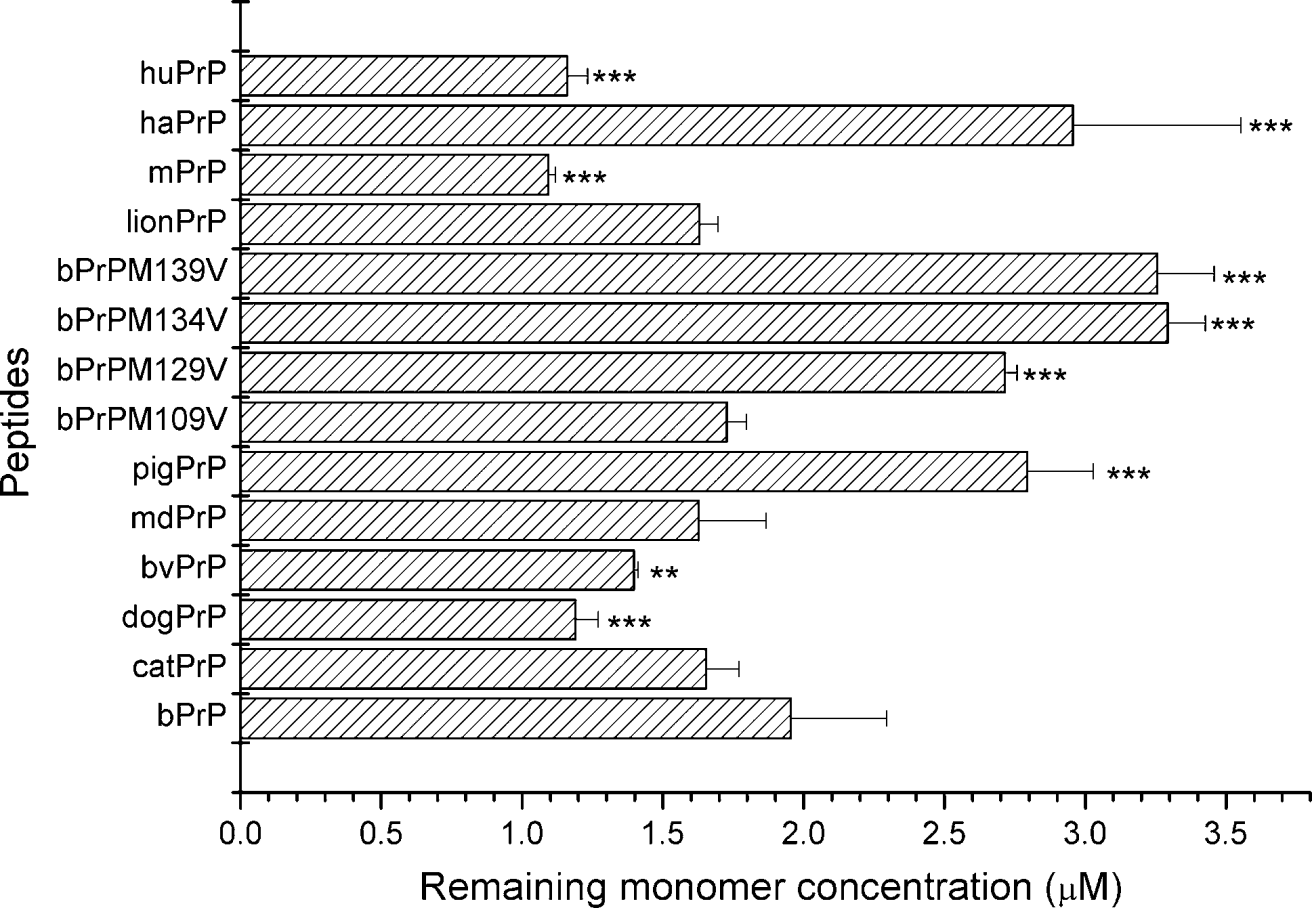
Remaining monomer concentration after fibril formation reaches equilibrium

The PrP peptides used in this study had good solubility in acidic buffer. CD spectroscopy showed that they were in a random coil state. In the structural ensemble, a small portion of the peptides is in the less stable “amyloid-precursor state” and, during prolonged incubation, the molecules in the “amyloid-precursor state” gradually associate and form a stable aggregation form, called nuclei. The nuclei associate with other molecules in the “amyloid-precursor state” one-dimensionally and finally form amyloid fibrils. The concentration of the peptide remaining in solution, also called the critical concentration, is used to represent the thermodynamic solubility of the peptide. The PrP peptides were dissolved at a concentration of 50 μM in 20 mM NaOAc, 140 mM NaCl (pH 3.7) and incubated at 25 °C for 3 weeks, then the fibrils in the solution were spun down and the concentration of peptide remaining in the supernatant determined by high-pressure liquid chromatography (HPLC). The thermodynamic solubility results are shown in Fig. 12 and are compared with the properties detailed in Table 2. Met is more hydrophilic than Val, Ile, and Leu, while Asn is more hydrophilic than Ser. Although the I139 → M substitution indeed increased the thermodynamic solubility of the peptide (bPrPI139M and haPrP), our results showed that the thermodynamic solubility of the peptide was not proportional to the sum of the hydrophilicity of its amino acid composition. For example, a comparison of bPrP and mPrP with exactly the same amino acid composition showed that their thermodynamic solubilities were significantly different. Moreover, thermodynamic solubility did not correlate with lag time or seeding efficiency.

## Conclusions

Amyloid formation is a special kind of protein aggregation, in which a special conformer (amyloid-precursor state) stacks regularly and the stacked aggregate promotes further association. During evolution, amino acid residues in mammalian prion peptides have mutated to others with similar chemical properties, with hydrophobic residues mutating to another hydrophobic residue (V, L, I, M) and hydrophilic residues mutating to another hydrophilic residue (N, S). However, some amino acid differences, such as V112M, N143S, and I139M, in prion peptides affect the population of the amyloid-precursor state and thus affect nucleation. Based on our results, we conclude that Met112 is an amyloid formation-promoting residue, while Ser143 and Met139 are amyloid formation-retarding residues. Moreover, the effect of one mutation on amyloidogenesis does not correlate with its effect on the seeding barrier. Using bPrP fibrils for seeding, the S135N (mule deer PrP) and N143S (pig PrP) mutations led to the lowest heterogeneous seeding efficiency (8-fold decrease) and the M129L (dog PrP) and I139M mutations led to a fourfold decrease in seeding efficiency, but interestingly, L138M (bank vole PrP) slightly increased seeding efficiency. Finally, when residues with opposite effects are present in the same peptide, their effects tend to cancel each other out.

## Electronic supplementary material

Below is the link to the electronic supplementary material.

Figure S1. CD spectra of huPrP peptide seeded with bPrP seed at different incubation times; Figure S2. CD spectra of huPrPM129V peptide seeded with bPrP seed at different incubation times.

Supplementary material 1 (PDF 184 kb)
