# Supplementary material for: How do amino acid substitutions affect the amyloidogenic properties and seeding efficiency of prion peptides
Source: Amino Acids. 2013 Jun 5;45(4):785–96. doi: 10.1007/s00726-013-1522-0 (PMC3776267; doi:10.1007/s00726-013-1522-0)
Supplement: Supplementary file 1 — Supplementary material 1 (PDF 184 kb) [file 726_2013_1522_MOESM1_ESM.pdf]

How do amino acid substitutions affect the amyloidogenic properties and seeding efficiency of prion peptides

Amino Acids

Chi-Chen Chuang[†‡], Tai-Yan Liao[‡], Eric H.-L Chen[‡], Rita P.-Y. Chen[†‡]

[†]Department of Biochemical Science and Technology, National Taiwan University, Taipei 106, Taiwan, R. O. C.
[‡]Institute of Biological Chemistry, Academia Sinica, Taipei 115, Taiwan, R. O. C.

*** Author to whom correspondence should be addressed:**
Rita P.-Y. Chen
Institute of Biological Chemistry, Academia Sinica, No 128, Sec 2, Academia Rd, Nankang, Taipei, 115, Taiwan
Tel: +(886)-2-2785-5696; Fax: +(886)-2-2788-9759; E-mail: pyc@gate.sinica.edu.tw

Supplementary material

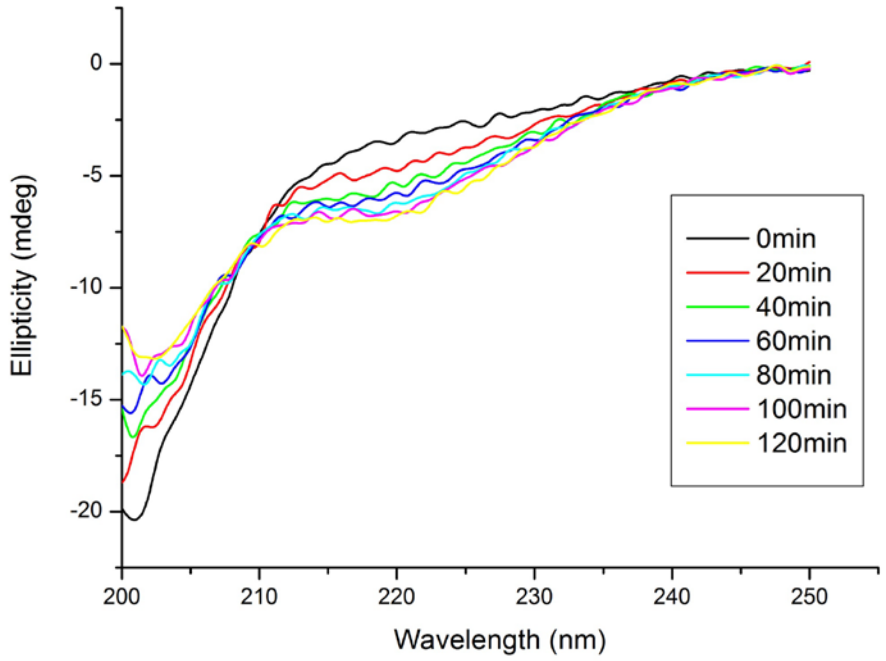

Figure S1. CD spectra of huPrP peptide seeded with bPrP seed at different incubation times. HuPrP (50 μM) dissolved in 20 mM NaOAc, 140 mM NaCl (pH 3.7) was seeded with 50 μL of the bPrP seed. Spectral changes indicate amyloid fibril formation.

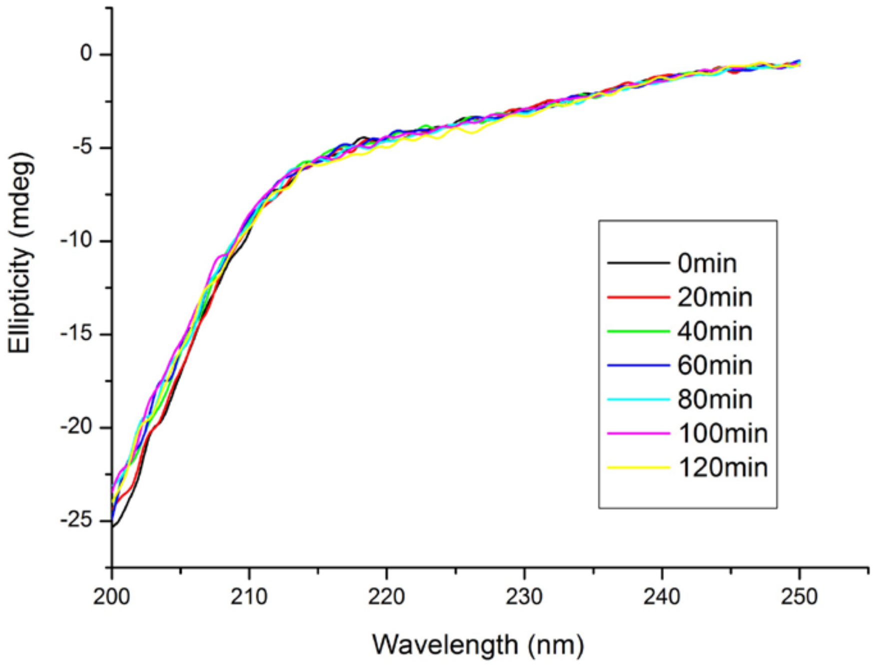

Figure S2. CD spectra of huPrPM129V peptide seeded with bPrP seed at different incubation times. HuPrPM129V (50 μM) dissolved in 20 mM NaOAc, 140 mM NaCl (pH 3.7) was seeded with 50 μL of the bPrP seed. Due to seeding barrier, no fibril propagation could be recorded. The spectra indicate no scattering artifacts due to protein aggregation during observation.
